# 

**Published:** 1892-10

**Authors:** 


					﻿Vol XII.
OCTOBER, 1892.
ND. 10.
JBHE
fJOMCEOPATHlC pHY^lClAfl,
A Monthly Journal of Homoeopathic Materia Medica
and Clinical Medicine.
•* He is a freeman whom truth makes free, and all are slaves betide.”
“ Seek the Truth : come whence it may, cost what it will.”
EDITED AND PVB1J8HED BY
WALTER ML JAMES, M. D.
PHILADELPHIA :
No. 112& spkucb: btrkkt.
LONDON:
ALFRED HEATH & CO., Sole Agents fob Great Britain,
114 Ebury Street, Eaton Square.
t^Jearly Subscription, S2.SO. invariably in advonee. Single numbers, 24 ets.
Entered at the Philadelphia Peet-OSee aa Second Claae Mall Matter.
				

